# Spectrophotometric Methods for the Determination of Sitagliptin and Vildagliptin in Bulk and Dosage Forms

**Published:** 2011-03

**Authors:** Ramzia I. El-Bagary, Ehab F. Elkady, Bassam M. Ayoub

**Affiliations:** *Department of Pharmaceutical Chemistry, Faculty of Pharmacy, Cairo University, Kasr El-Aini St., Cairo, Egypt*

**Keywords:** vildagliptin, sitagliptin phosphate, spectrophotometry, charge transfer reaction, pharmaceutical preparation

## Abstract

Simple, accurate and precise spectrophotometric methods have been developed for the determination of sitagliptin and vildagliptin in bulk and dosage forms. The proposed methods are based on the charge transfer complexes of sitagliptin phosphate and vildagliptin with 2,3-dichloro-5,6-dicyano-1,4-benzoquinone (DDQ), 7,7,8,8-tetracyanoquinodimethane (TCNQ) and tetrachloro-1,4-benzoquinone (*p*-chloranil). All the variables were studied to optimize the reactions conditions. For sitagliptin, Beer’s law was obeyed in the concentration ranges of 50-300 μg/ml, 20-120 μg/ml and 100-900 μg/ml with DDQ, TCNQ and *p*-chloranil, respectively. For vildagliptin, Beer’s law was obeyed in the concentration ranges of 50-300 μg/ml, 10-85 μg/ml and 50-350 μg/ml with DDQ, TCNQ and *p*-chloranil, respectively. The developed methods were validated and proved to be specific and accurate for the quality control of the cited drugs in pharmaceutical dosage forms.

## INTRODUCTION

**Figure 1:**
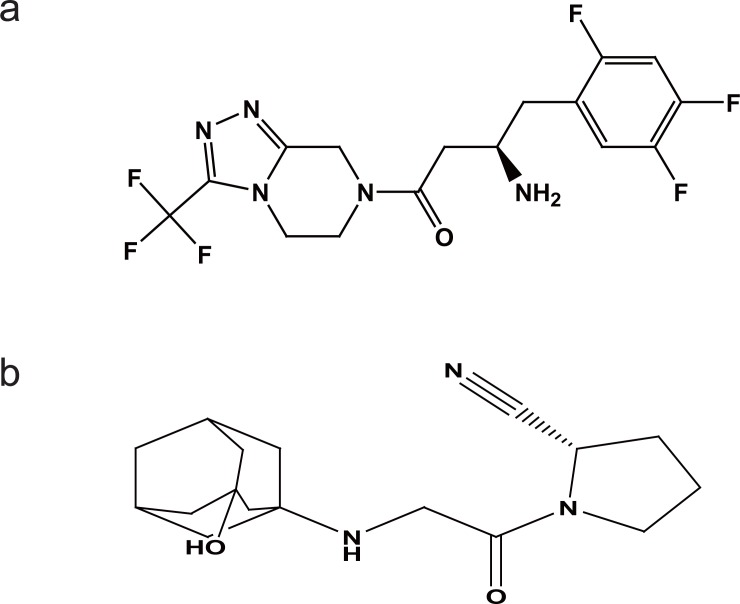
a, sitagliptin; b, vildagliptin.

Sitagliptin, 1,2,4-triazolo[4,3-a]pyrazine,7-[(3R)-3-amino-1-oxo-4-(2,4,5-trifluoro phenyl)butyl]-5,6,7,8-tetrahydro-3-(trifluoromethyl) phosphate (STG) (Fig. 1a) and vildagliptin, S-1-[N-(3-hydroxy-1-adamantyl)glycyl]pyrrolidine-2-carbonitrile (VDG) (Fig. 1b) are oral hypoglycemic drugs of the dipeptidyl peptidase-4(DPP-4) inhibitor class (1). DPP-4 inhibitors represent a new therapeutic approach to the treatment of type 2 diabetes that functions to stimulate glucose-dependent insulin release and reduce glucagons levels. This is done through inhibition of the inactivation of incretins, particularly glucagon-like peptide-1 (GLP-1) and gastric inhibitory polypeptide (GIP), thereby improving glycemic control (2).

Literature survey reveals that only one high performance liquid chromatographic (HPLC) method has been developed for the determination of STG in plasma (2). A high turbulence liquid chromatographic (HTLC) method for the determination of STG in human urine has been developed (3). It is worth noting that only one method has been adopted for the determination of STG in its pharmaceutical formulation. This method was based on colorimetric determination of STG after its reaction with formaldehyde and acetylacetone (1).

Spectrophotometry continues to be very popular, because of its simplicity, versatility and low cost. This study represents the first charge transfer complexation methods for the determination of STG and VDG in bulk and pharmaceutical formulations. Charge transfer reactions have been widely used for the determination of electron donating compounds through interaction with π-acceptors. Among the electron acceptors mostly used in literature are 2,3-dichloro-5,6-dicyano-1,4-benzoquinone (DDQ) (4-7), 7,7,8,8tetracyanoquinodimethane (TCNQ) (8-11), tetrachloro-1,4-benzoquinone (*p*-chloranil) (12-14).

## EXPERIMENTAL

### Instrumentation

A Jenway 6800 double beam ultraviolet/visible spectrometer connected to an IBM compatible computer with 1-cm quartz cell and supported with Jenway flight deck software was used.

### Reagents and reference samples

DDQ, TCNQ and *p*-chloranil were supplied from Sigma Aldrich Chemie GmbH (Steinheim, Germany). Freshly prepared solutions were prepared (DDQ solution a: 260.3 mg/100 ml in methanol, solution b: 336.7 mg/100 ml in acetonitrile), (TCNQ solution a: 171.1 mg/100 ml, solution b: 134.6 mg/100 ml both in methanol), (*p*-chloranil solution a: 939.7 mg/100 ml, solution b: 486.3 mg/100 ml both in dimethylformamide). Pharmaceutical grade sitagliptin phosphate monohydrate, certified to contain 99.80% and Januvia^®^ tablets nominally containing 128.5 mg of sitagliptin phosphate monohydrate per tablet (batch no. S0273) were kindly supplied from Merck Sharp and Dohme Co. (Cairo, Egypt). Pharmaceutical grade vildagliptin, certified to contain 99.70% and Galvus^®^ tablets nominally containing 50 mg per tablet (batch no. V6498) were kindly supplied from Novartis Europharm limited company (London, U.K.). Standard stock solutions of each drug (1 mg/ml) were prepared by dissolving 100 mg of the drug in the selected solvent and completing the volume to 100 ml in a volumetric flask. All the solvents used were of analytical grade.

### General procedures and calibration graphs

**Method using DDQ.** Aliquots of STG and VDG containing (0.5–3 mg) were transferred into two separate sets of 10 ml volumetric flasks, treated with 1 ml DDQ solution (a) and solution (b), respectively and allowed to stand for 20 min and 30 min, respectively at room temperature (20–25°C) and diluted to volume with methanol and acetonitrile, respectively. The absorbance was measured at 461 nm and 486 nm, respectively against reagent blank.

**Method using TCNQ.** Aliquots of STG containing (0.2–1.2 mg) and VDG containing (0.1–0.85 mg) were transferred into two separate sets of 10 ml volumetric flasks, treated with 1 ml TCNQ solution (a) and solution (b), respectively and allowed to stand for 30 min at room temperature (20–25°C) and diluted to volume with methanol. The absorbance was measured at 837 nm and 838 nm, respectively against reagent blank.

**Method using *p*-chloranil.** Aliquots of STG containing (0.5–9 mg) and VDG containing (0.5–3.5 mg) were transferred into two separate sets of 10 ml volumetric flasks, treated with 1 ml *p*-chloranil solution (a) and solution (b), respectively and allowed to stand for 40 min and 10 min, respectively at room temperature (20–25°C) and diluted to volume with dimethylformamide. The absorbance was measured at 555 nm against reagent blank.

### Procedure for the assay of the tablets

Twenty tablets of each drug were weighed and the coats in case of STG were removed by carefully rubbing with a clean tissue wetted with using methanol. An accurately weighed amount of the finely powdered tablets equivalent to 100 mg of each drug was made up to 100 ml with the selected solvent, the solution was filtered. The procedure was continued as mentioned under general procedures and calibration graphs.

### Effect of the amount of the reagent

Aliquots of STG (1.9 × 10^-3^ M) and VDG (3.3 × 10^-3^ M) stock solutions were introduced into two separate sets of 10 ml volumetric flasks. Different aliquots of (DDQ, TCNQ and *p*-chloranil) were added to each flask to obtain different drug/reagent molar ratios in an increasing order, and then the procedure was continued as mentioned under general procedures and calibration graphs.

### Stoichiometric relationship

Job’s method of continuous variation was employed, between standard solutions of each drug (1.9 × 10^-3^ M of STG and 3.3 × 10^-3^ M of VDG) with different reagents (DDQ, TCNQ and *p*-chloranil). The concentration of each reagent was adjusted to be 1.9 × 10^-3^ M in the stoichiometric study of STG and to 3.3 × 10^-3^ M in the stoichiometric study of VDG. A series of solutions was prepared in which the total volume of the drug and the reagent was kept at 5 ml. The method was continued as mentioned under the general procedures for the calibration graphs.

## RESULTS AND DISCUSSION

### Formation of the charge transfer complexes

**Figure 2:**
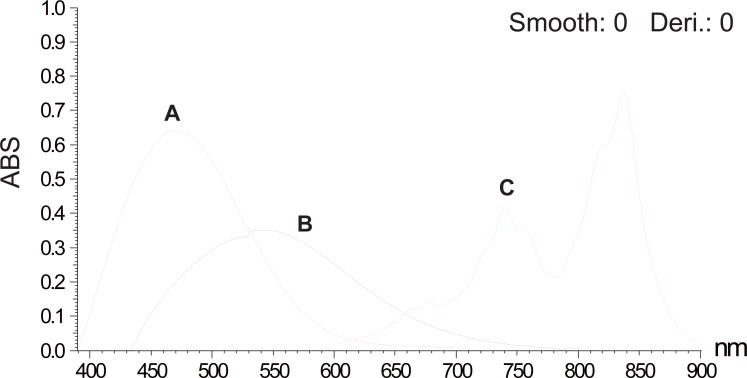
Absorption spectra for the reaction products of 300 μg/ml of sitagliptin with DDQ solution a (A) and with *p*-chloranil solution a (B) and for the reaction product of 100 μg/ml of sitagliptin with TCNQ solution a (C).

**Figure 3:**
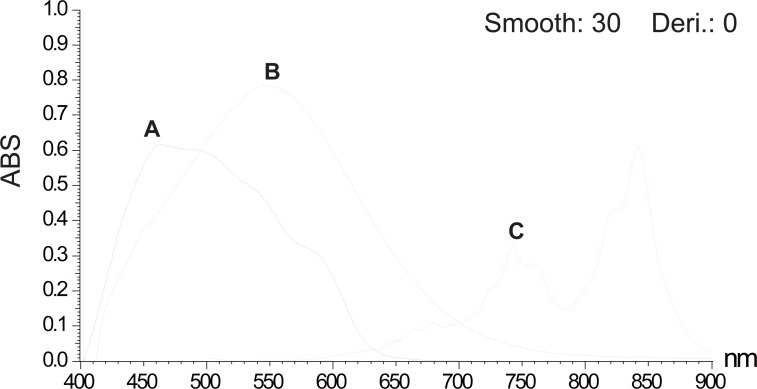
Absorption spectra for the reaction products of 150 μg/ml of vildagliptin with DDQ solution b (A), 350 μg/ml of vildagliptin with *p*-chloranil solution b (B) and for the reaction product of 50 μg/ml of vildagliptin with TCNQ solution b (C).

The charge transfer reagents applied in this work are DDQ, TCNQ and *p*-chloranil. DDQ is an electron deficient molecule due to the electron withdrawing effect of the two cyano and the two chloro groups. The reaction between DDQ as a π-acceptor with several drugs (4-7) to give a complex which dissociates in polar solvents to a highly colored radical anion has been reported. Likewise, TCNQ is a well known π-acceptor (8-11); this character is derived from the high electron affinity of polyene system conferred by the electron withdrawing effect of the four cyano groups and also from the planarity and high symmetry of TCNQ. Besides, primary, secondary and tertiary amines, both aliphatic and aromatic were shown to react with *p*-chloranil to produce a blue to purple colour. It was reported that *p*-chloranil could react with amines to form either mono substituted aminoquinones or radical ion pairs. It appears from literature survey that the reaction of *p*-chloranil with different drugs may differ according to the structure of the drug, solvent used and temperature (12-14). Different parameters affecting the reactions such as amount of the reagent, reaction time and stability of the color have been investigated. λ_max_ of measurements of STG and VDG with the three reagents are shown in Table 2 and Table 3. The absorption spectra of the reaction products of STG and VDG with the three reagents are shown in Figure 2 and Figure 3.

### Effect of solvent

Although many solvents have been used to carry out the charge transfer complex reactions, using the appropriate solvent in the described work was critical and might be different from one drug to another (Table 1 and Table 2). STG is soluble in dimethylformamide and methanol but it is practically insoluble in acetonitrile and most of organic solvents. Higher sensitivity of the reactions was attained in methanol for DDQ and TCNQ methods and in dimethylformamide for *p*-chloranil method. For VDG, it is freely soluble in most of the organic solvents but the reaction with TCNQ was not quantitative in acetonitrile. Thus methanol in this reaction was appropriate and quantitative. Higher sensitivity of the reactions was attained in acetonitrile, methanol and dimethylformamide for DDQ, TCNQ and *p*-chloranil, respectively.

**Table 1:** Results obtained by the proposed methods for the determination of Sitagliptin phosphate using DDQ, TCNQ and *p*-chloranil

Item	DDQ method	TCNQ method	*P*-chloranil

Solvent	methanol	methanol	dimethylformamide
Time of reaction	10 minutes	20 minutes	30 minutes
Stability of the color	60 minutes	50 minutes	70 minutes
λ_max_ of measurements	461 nm	837 nm	555 nm
Obedience of Beer’s law	50-300 μg/ml	20-120 μg/ml	50-900 μg/ml
Regression equation	A_461_=0.0032 × Conc._(μg/ml)_ - 0.0413	A_837_=0.0089 × Conc._(μg/ml)_ + 0.0061	A_555_=0.0012 × Conc._(μg/ml)_ + 0.0346
Regression coefficient (r^2^)	0.9992	0.9994	1.0
LOD μg/ml	9.79	3.26	1.40
LOQ μg/ml	29.67	9.89	4.25
S_b_	5.53 × 10^-5^	1.1 × 10^-4^	2 × 10^-6^
S_a_	4.08 × 10^-4^	8.58 × 10^-3^	1.15 × 10^-3^
Confidence limit of the slope	0.0032 ± 9.41×10^-4^	0.0089 ±1.98×10^-2^	0.0012 ± 2.65×10^-3^
Confidence limit of the intercept	-0.0413 ± 1.28 × 10^-4^	0.0061 ± 2.54 × 10^-4^	0.0346 ± 4.61 × 10^-6^
Standard error of the estimation	0.01118	9.22 × 10^-3^	1.24 × 10^-3^
Results			
1. Drug in bulk	100.19 ± 1.02	99.83 ± 1.33	99.66 ± 1.40
2. Drug in dosage form	98.84 ± 0.56	99.51 ± 1.56	99.53 ± 1.1
3. Drug added	100.16 ± 1.81	99.92 ± 1.43	100.33 ± 0.95

**Table 2:** Results obtained by the proposed methods for the determination of vildagliptin using DDQ, TCNQ and *p*-chloranil

Item	DDQ method	TCNQ method	*P*-chloranil

Solvent	acetonitrile	Methanol	dimethylformamide
Time of reaction	20 minutes	20 minutes	5 minutes
Stability of the color	60 minutes	70 minutes	60 minutes
λ_max_ of measurements	486 nm	838 nm	555 nm
Obedience of Beer’s law	50-300 μg/ml	10-85 μg/ml	50-350 μg/ml
Regression equation	A_486_ = 0.0040 × Conc_(μg/ml)_ - 0.0478	A_838_ = 0.0139 × Conc_(μg/ml)_ + 0.0392	A_555_ = 0.0021 × Conc_(μg/ml)_ + 0.0204
Regression coefficient (r^2^)	0.9998	0.9999	0.9998
LOD μg/ml	5.1	0.88	5.17
LOQ μg/ml	15.5	2.65	15.66
S_b_	5.36 × 10^-5^	6.44 × 10^-5^	3.8 × 10^-5^
S_a_	1.04 × 10^-2^	3.47 × 10^-3^	9.1 × 10^-3^
Confidence limit of the slope	0.0040 ± 2.4 × 10^-2^	0.0139 ± 8 × 10^-3^	0.0021 ± 2.1 × 10^-2^
Confidence limit of the intercept	-0.0478 ± 1.24 × 10^-4^	0.0392 ± 1.5 × 10^-4^	0.0204 ± 8.76 × 10^-5^
Standard error of the estimation	0.01122	4.04 × 10^-3^	7.94 × 10^-3^
Results			
1. Drug in bulk	99.58 ± 1.17	100.32 ± 1.00	99.3 ± 1.52
2. Drug in dosage form	100.09 ± 1.10	100.13 ± 0.41	99.73 ± 0.84
3. Drug added	100.32 ± 1.47	99.95 ± 1.33	100.2 ± 0.86

### Rate of complex formation and stability of the formed complex

A study of the effect of time revealed that the maximum color intensity in case of STG was attained at least after 10 min, 20 min and 30 min and remained stable for further 60 min, 50 min and 70 min with DDQ, TCNQ and *p*-chloranil, respectively (Table 1). For VDG, the color was attained at least after 20 min with DDQ, TCNQ methods and after 5 min with *p*-chloranil method and remained stable for further 60 min with DDQ and *p*-chloranil methods and for further 70 min with TCNQ method (Table 2).

### Sensitivity, Stoichiometric relationship and the amount of reagent

**Figure 4:**
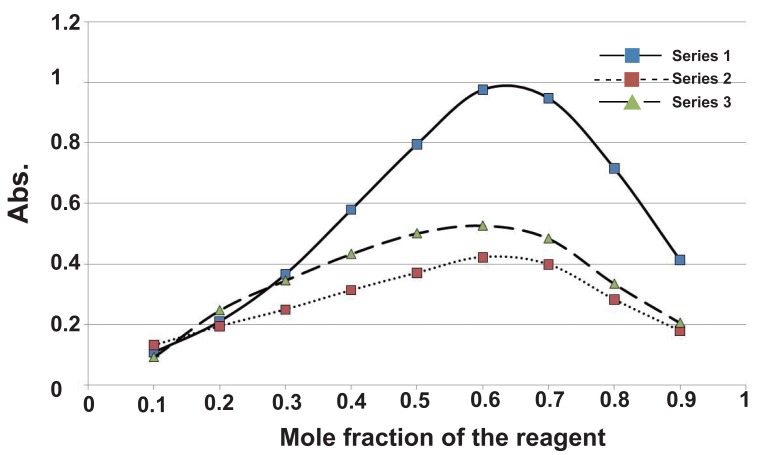
Continuous variation plot of the reaction products of sitagliptin with DDQ (series 1), TCNQ (series 2) and *P*-chloranil (series 3).

**Figure 5:**
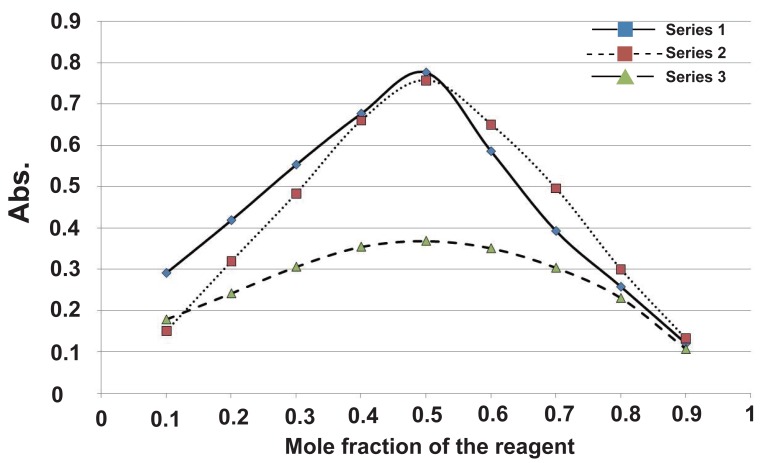
Continuous variation plot of the reaction products of vildagliptin with DDQ (series 1), TCNQ (series 2) and *P*-chloranil (series 3).

Job’s method of continuous variation revealed a molar ratio of (1:2) drug: reagent in the case of STG with the three reagents (Fig. 4). This may be attributed to relative availability of the lone pair of the primary amine along with the lone pair of N3 of the triazole ring, For VDG, a molar ratio of (1:1) drug: reagent was obtained with the three reagents (Fig. 5) This may be attributed to the availability of one center as an electron donating group which is the secondary amine while the other tertiary amine is less available as it is consumed by resonance.

### Quantification, accuracy and precision

Standard calibration curves were prepared by separately preparing series of different concentrations of the two drugs and applying the suggested procedures with DDQ, TCNQ, and *p*-chloranil. Beer’s law was valid within microgram concentration range of STG (Table 1) and VDG (Table 2). The linearity of the calibration curves were validated by the high value of correlation coefficients. The analytical data of the calibration curves including standard deviations for the slope and intercept (S_b_, S_a_) are summarized in Table 2 and Table 3. The regression equations of these calibration graphs were utilized for determination of concentrations of the cited drugs in laboratory prepared mixtures and tablets. The reproducibility and accuracy of the suggested methods were assessed using different laboratory prepared solutions of different concentrations and determination of the concentrations in tablets. The results obtained were of good accuracy and precision. The applicability of the procedures for estimation of tablets was validated using standard addition technique as a check for accuracy (Table 1 and Table 2).

**Table 3:** Statistical comparison between the results of the proposed spectrophotometric methods and the official methods for the determination of sitagliptin

Statistical term	Reference Method^b^	DDQ method	TCNQ method	*p*-Chloranil mehtod

Mean	100.5	100.19	99.83	99.66
S.D. ±	1.39	1.02	1.33	1.4
S.E. ±	0.62	0.46	0.6	0.63
% RSD	1.39	1.02	1.33	1.40
n	5	5	5	5
V	1.93	1.04	1.77	1.96
t (a2.306)		0.402	0.779	0.952
F (a6.39)		1.86	1.09	0.98

a Figures in parentheses are the theoretical t and F values at (*p*=0.05);

b Reference methods: aliquots of standard solutions in distilled water containing 2-10 μg/ml STG were measured using water as a blank. The amount of STG present in the samples were computed from the corresponding calibration curves (1). For VDG, aliquots of standard solutions in distilled water containing 5-25 μg/ml were measured using water as a blank. The amount of VDG present in the samples were computed from the corresponding calibration curves.

**Table 4:** Statistical comparison between the results of the proposed spectrophotometric methods and the official methods for the determination of vildagliptin

Statistical Term	Reference Method^b^	DDQ method	TCNQ method	*p*-Chloranil method

Mean	100.01	99.58	100.32	99.3
S.D. ±	0.99	1.17	1	1.52
S.E. ±	0.44	0.52	0.45	0.68
% RSD	0.99	1.17	1	1.52
n	5	5	5	5
V	0.98	1.37	1	2.31
t (a2.306)		0.627	0.493	0.875
F (a6.39)		0.72	0.98	0.42

a Figures in parentheses are the theoretical t and F values at (*p*=0.05);

b Reference methods: aliquots of standard solutions in distilled water containing 2-10 μg/ml STG were measured using water as a blank. The amount of STG present in the samples were computed from the corresponding calibration curves (1). For VDG, aliquots of standard solutions in distilled water containing 5-25 μg/ml were measured using water as a blank. The amount of VDG present in the samples were computed from the corresponding calibration curves.

A statistical analysis of the results obtained by the proposed methods for the determination of STG and VDG was carried out by “SPSS statistical package version 11”. The significant difference between groups were tested by one way ANOVA (F-test) at *p*=0.05 as shown in Table 3 and Table 4. The test ascertained that there was no significant difference among the methods.

## CONCLUSION

The proposed spectrophotometric methods have the advantages of simplicity, precision, accuracy and convenience for the quantitation of STG and VDG. Hence, the proposed methods can be used for the quality control of the cited drugs and can be extended for routine analysis of the drugs in bulk and dosage forms.
